# Supplementary material for: Targeted DNA Sequencing of Cutaneous Melanoma Identifies Prognostic and Predictive Alterations
Source: Cancers (Basel). 2024 Mar 29;16(7):1347. doi: 10.3390/cancers16071347 (PMC11011039; doi:10.3390/cancers16071347)
Supplement: Supplementary file 1 [file cancers-16-01347-s001.zip › Supplementary.pdf]

Supplementary Figures.

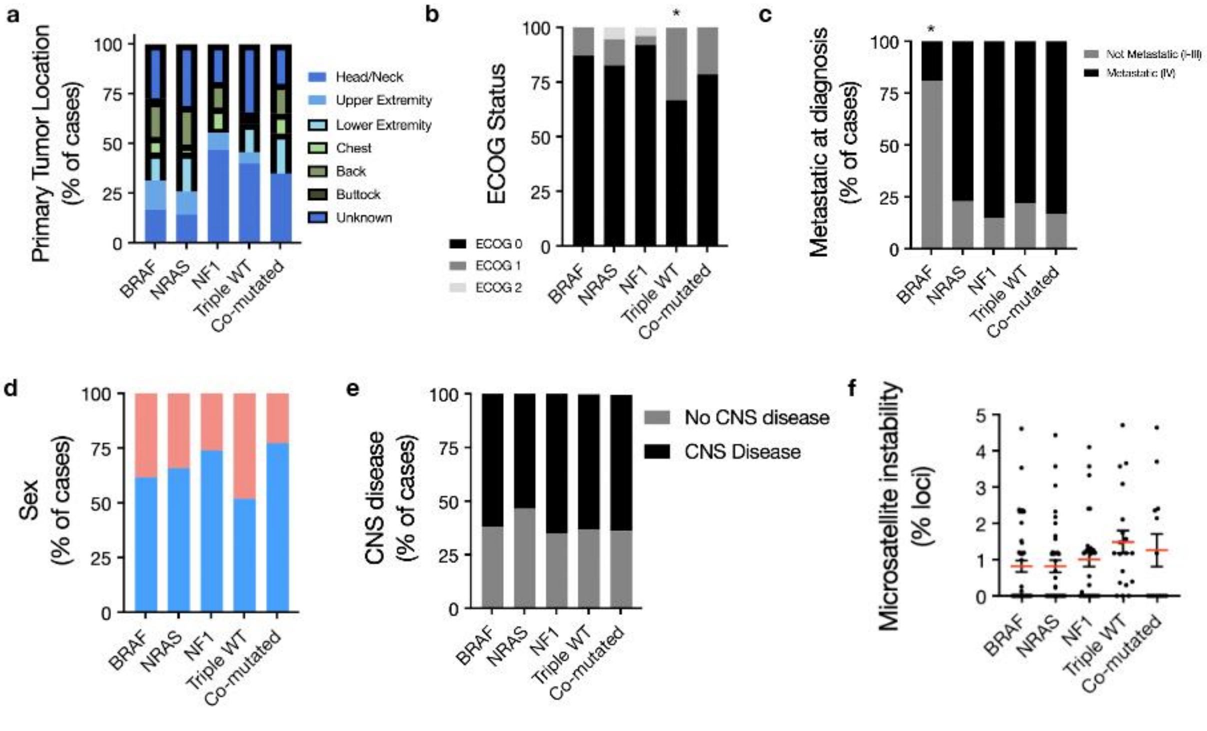

Figure S1. Baseline clinical correlations based on molecular group. a. Location of primary tumor based on molecular driver. *NF1* mutant, co-mutated and triple wild type tumors were more likely to occur on the head/neck. b. There were no statistically significant differences in sex breakdown between molecular subgroups. c. *BRAF* mutation was significantly associated with non-metastatic presentation at diagnosis (chi-square test $p < 0.0001$) . d. TWT tumors were significantly more likely to present as ECOG 1+ (n=7/21, 33%) compared with non TWT tumors (n=21/146, 15%).; p = 0.033. e. There were no statistically significant differences in rates of development of CNS disease between TCGA driver groups. f. There were no statistically significant differences in microsatellite instability (MSI) between molecular driver groups.

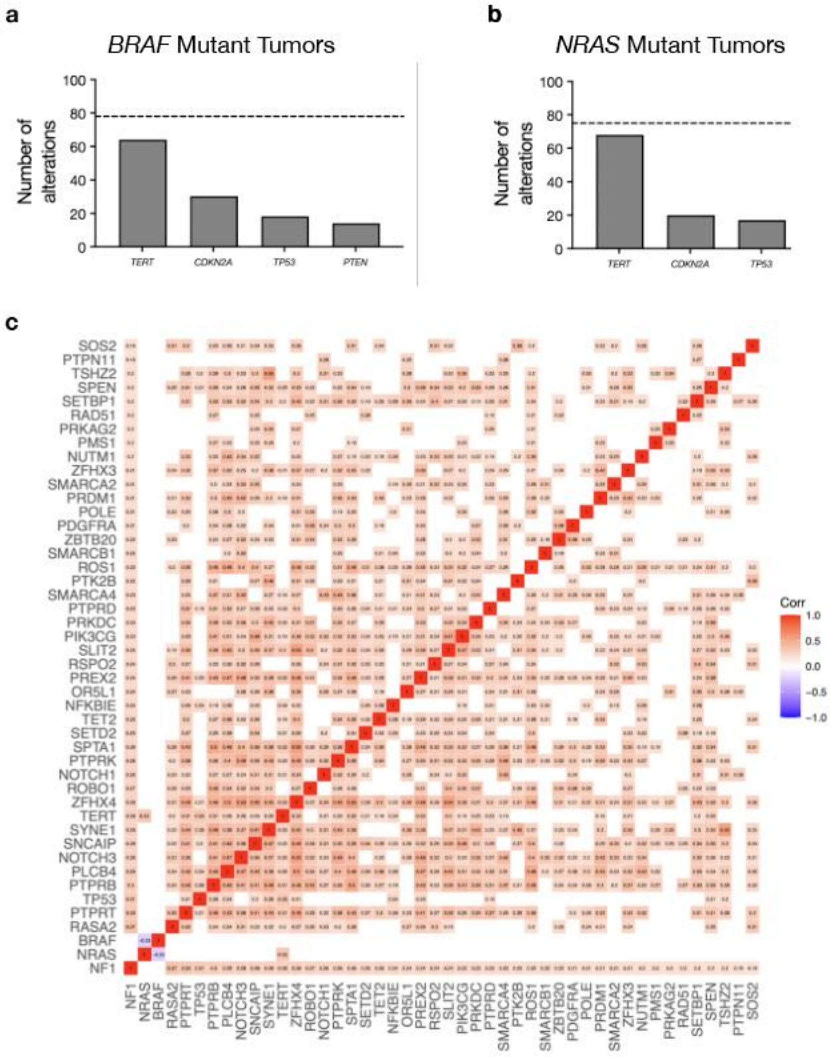

Figure S2. Analysis of co-mutation patterns. a. Most frequently co-altered genes in *BRAF* mutant tumors. b. Most frequently co-altered genes in *NRAS* mutant tumors. c. Correlation matrix summarizing statistically significant gene correlations across the entire cohort without imposing a strict Pearson correlation coefficient threshold.

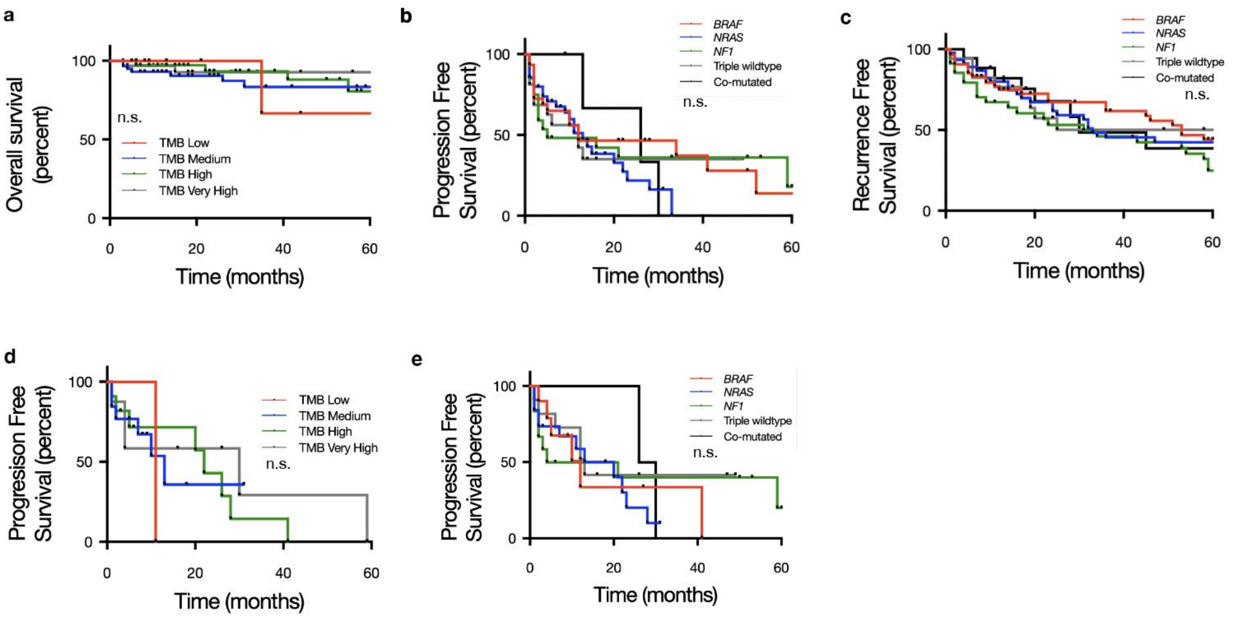

Figure S3. Clinical outcomes based on molecular signatures and treatment. a. Kaplan Meier (KM) curve depicting overall survival stratified by TMB group which showed no statistically significant differences in overall survival outcomes for all 254 patients included. b. KM curve depicting progression free survival on any first line systemic therapy based on TCGA molecular driver which showed no statistically significant differences between groups. c. KM curve depicting recurrence free survival following resection based on TCGA molecular driver. There were no statistically significant differences in RFS between driver groups. d. KM curve depicting progression free survival on anti-PD1 monotherapy stratified by TMB group. There were no statistically significant differences in PFS on anti-PD1 between these groups. e. KM curve depicting PFS on anti-PD1 monotherapy stratified by TCGA molecular driver. There were no statistically significant differences in PFS on anti-PD1 monotherapy between TCGA driver groups.

Supplementary Tables.

Supplementary Table 1. Compiled variant list from the UCSF500 assay, a CLIA certified, capture based targeted DNA sequencing assay of 529 cancer-associated genes.

Supplementary Table 2. Univariable and multivariable analysis for OS

Supplementary Table 3. Univariable and multivariable analysis for PFS on any first line immunotherapy.

Supplementary Table 4. Univariable and multivariable analysis for RFS

Supplementary Table 5. Univariable and multivariable analysis for PFS on single agent immune checkpoint inhibition.

Supplementary Table 6. Univariable and multivariable analysis for PFS on dual agent immune checkpoint inhibition.
